# Potential impact of unblinding on observed treatment effects in Alzheimer's disease trials

**DOI:** 10.1002/alz.13690

**Published:** 2024-02-21

**Authors:** Frank J. Wolters, Jeremy A. Labrecque

**Affiliations:** ^1^ Department of Epidemiology Erasmus MC – University Medical Centre Rotterdam Rotterdam the Netherlands; ^2^ Department of Radiology & Nuclear Medicine Erasmus MC – University Medical Centre Rotterdam Rotterdam the Netherlands

**Keywords:** Alzheimer's disease, amyloid, epidemiology, randomized controlled trial, unblinding

## Abstract

**INTRODUCTION:**

Adverse effects of monoclonal antibodies against amyloid beta are common, and may affect validity of randomized controlled trials (RCTs) through unblinding of participants.

**METHODS:**

We used observations from published phase 3 RCTs in Alzheimer's disease to calculate the magnitude of unblinding effects on cognition that would be required to explain observed cognitive benefits in RCTs.

**RESULTS:**

In trials of lecanemab, aducanumab, and donanemab, incidence of amyloid‐related imaging abnormalities with active treatment ranged from 22% to 44%, the vast majority of which presumably led to unblinding. Effects of unblinding on the Clinical Dementia Rating Sum of Boxes required to fully explain observed drug effects ranged from 1.1 point (95% confidence interval: 0.2–2.0) with aducanumab, to 3.3 points (2.1–4.4) with donanemab and 3.7 points (2.0–5.6) with lecanemab. Infusion‐related reactions were common, with potential unblinding effects particularly for lecanemab. Similar patterns were observed for the Alzheimer's Disease Assessment Scale Cognitive subscale.

**DISCUSSION:**

Psychological treatment effects due to unblinding may explain a substantial share of observed treatment effects in RCTs.

## 1 BACKGROUND

Recent randomized controlled trials (RCTs) have shown that treatment with monoclonal antibodies against amyloid beta (Aβ) reduces the rate of cognitive decline in patients with Alzheimer's disease (AD), compared to placebo. However, internal validity of RCTs too is susceptible to methodological caveats, and AD trials are notably susceptible to attrition and potential unblinding of participants due to side effects. Active treatment with monoclonal antibodies is often accompanied by side effects, with amyloid‐related imaging abnormalities (ARIA) occurring in 22% to 44% of patients, and infusion‐related reactions reported in up to 26% of patients.^1^, ^2^, ^3^ When these side effects give rise to symptoms or changes in the treatment regimen, they could lead to unblinding of study participants to their allocated treatment. Insight in treatment allocation may influence outcome measures of cognition, thereby hampering validity of the estimated treatment efficacy. This is an important caveat to consider when determining clinical relevance of treatment effects in RCTs against AD, particularly when weighing the risk to benefit ratio, and costs of intervention. Several researchers have raised concerns about the potential effects of unblinding in these trials,^4^, ^5^ but no published studies have investigated whether unblinding in RCTs can realistically explain the observed clinical differences between active treatment and placebo.

Using results of prior RCTs in a simulation study, we aimed to determine whether unblinding for treatment allocation due to adverse effects can account for the efficacy of monoclonal antibodies against Aβ in patients with AD.

## 2 METHODS

### 2.1 Trial eligibility

We searched the PubMed library and Alzforum.org for trial publications regarding the clinical efficacy of monoclonal antibodies against AD. We selected all phase 3 RCTs that reported statistically significant positive effects of active treatment, compared to placebo, on the primary outcome measure.

### 2.2 Data extraction

From published results of all trials, we extracted the following data: study characteristics (i.e., sample size, follow‐up duration), patient characteristics (entry diagnosis, Mini‐Mental State Examination at baseline, age, sex, race), and trial results (cognitive outcomes in active treatment and placebo arms, incidence of adverse events including ARIA‐H [hemorrhagic], and ARIA‐E [edema], and infusion‐related reactions). We also reviewed the protocol of each trial for management of adverse events and side effects. If any of the required data were not available in the original trial publications, we searched the internet for press releases and additional reports or presentations of trial results.

### 2.3 Statistical analysis

Extracted data were summarized and presented per included trial. As the duration of the included trials was similar (i.e., 18 months), we used the reported cumulative incidences of adverse events rather than calculating incidence rates. All three trials reported outcome data for the Clinical Dementia Rating Sum of Boxes (CDR‐SB) and Alzheimer's Disease Assessment Scale Cognitive subscale (ADAS‐Cog). We primarily looked at the CDR‐SB as this was the main outcome measure in two of the three trials, and report results on the ADAS‐Cog to enhance comparison and robustness of findings. For TRAILBLAZER‐ALZ‐2, we extracted incidence of “treatment‐emergent adverse event[s]”, not counting adverse events noted on safety magnetic resonance imaging (MRI), to avoid overestimation of ARIA impact.^3^

We defined the measured cognition as a function of the treatment effect and, potentially, therapeutic insight inferred from the presence of adverse events:

$$E\;\left[Y\right]=\;\alpha +\beta_{Tx}*Tx+\beta_{AE}*AE+\varepsilon$$

where *Y* is the cognitive outcome measure, α is an intercept, β_Tx_ the treatment effect, *Tx* a treatment indicator (0 = placebo; 1 = active treatment), β_AE_ the effect of adverse events on the outcome, and *AE* an indicator of adverse events (0 or 1). The average value of the outcome in each arm is:

$$E\;\left[Y|Tx\;=\;1\right]=\;\alpha +\beta_{Tx}+\beta_{AE}*P\left(AE_{1}\right)+\varepsilon_{1}$$

$$E\;\left[Y|Tx\;=\;0\right]=\;\alpha +\beta_{AE}*P\left(AE_{0}\right)+\varepsilon_{0}$$

**RESEARCH IN CONTEXT**

1. **Systematic review**: The authors reviewed the PubMed library for trial publications and comments thereon regarding the clinical efficacy of monoclonal antibodies against Alzheimer's disease (AD). Despite extensive debate on adverse treatment effects, they found no reports quantifying the potential impact of unblinding. Relevant citations discussing prior recommendations for trial design and monitoring are appropriately cited.
2. **Interpretation**: Adverse effects of monoclonal antibodies against AD often unblind participants of randomized controlled trials (RCTs) to treatment allocation. Unblinding is unlikely to fully account for observed effects of lecanemab and donanemab on the Clinical Dementia Rating Sum of Boxes and Alzheimer's Disease Assessment Scale Cognitive subscale, but may explain a significant share of the observed treatment effects for all drugs, including aducanumab.
3. **Future directions**: Adaptations in trial design and more detailed reporting of unblinding events could aid in quantifying and limiting the impact of unblinding in RCTs. Meanwhile, the estimates from this study warrant consideration when interpreting clinical relevance of the investigated therapies.

Note that the proportion of adverse events in the treatment arm, $P(AE_{1})$, is not the same as in the control arm, $P(AE_{0})$. The difference in the outcome between the two treatment groups is:

$$E\left[Y|Tx\;=\;1\right]-E\;\left[Y|Tx\;=\;0\right]=\beta_{Tx}\;+\beta_{AE}*\left(P\left(AE_{1}\right)-P\left(AE_{0}\right)\right)$$

Assuming $\beta_{AE}=\;0$, that is, that having an adverse event has no effect on the outcome, the difference in the treatment groups is entirely due to $\beta_{Tx}$. We wish to explore the different, possibly extreme, possibility that the biological effect of treatment on *Y* is 0 and that the entirety of the observed difference in cognitive outcome is due to the effect of therapeutic insight and the difference in the proportion of participants with adverse events. This assumes that participants with no adverse events have no therapeutic insight and that all participants with adverse events have complete therapeutic insight. If this is the case, the β_AE_ needed to explain the difference between the treatment groups is:

$$\beta_{AE}=\frac{E\left[Y|Tx=1\right]-E\left[Y|Tx=0\right]}{P\left(AE_{Tx=1}\right)-P\left(AE_{Tx=0}\right)}\;$$

Therefore, using the values for the observed treatment effect, E[Y|Tx=1]−E[Y|Tx=0], and the difference in the proportion of participants experiencing adverse events in the treatment and control arms, we can calculate how much effect therapeutic insight would need to have on cognitive outcomes to explain the entire difference in the observed outcome between treatment arms. The resulting parameter $\beta_{AE}$ therefore reflects the magnitude of the effect that unblinding would need to have on the cognitive outcome measure (i.e., CDR‐SB or ADAS‐Cog), to fully explain away the observed difference between active treatment and placebo in the respective trials. We further express estimates relative to the standard deviation of the outcome measure (Cohen *d*). Results were truncated at the maximum possible score of the CDR‐SB and ADAS‐Cog assessments.

## 3 RESULTS

We identified three trials that reported statistically significant differences in change in cognition between active treatment and placebo groups, that is, the CLARITY‐AD trial of lecanemab, the EMERGE trial of high‐dose aducanumab, and the TRAILBLAZER‐ALZ‐2 trial of donanemab. Key features of these trials are presented in Table 1. The CDR‐SB was the primary outcome for CLARITY‐AD and EMERGE, and a secondary outcome for TRAILBLAZER‐2. Slightly larger effects on the CDR‐SB were observed for active treatment in TRAILBLAZER‐ALZ‐2, compared to treatment effects in CLARITY‐AD and EMERGE. Decline within the placebo group was also somewhat larger in TRAILBLAZER‐AL2 than in the other two trials. Trial results were similar among all trials with respect to decline on the ADAS‐Cog (Table 1).

**TABLE 1:** Key characteristics of included randomized controlled trials.

	CLARITY‐AD	EMERGE	TRAILBLAZER‐ALZ‐2
Drug name	Lecanemab	Aducanumab	Donanemab
Sample size	1734	1638^a^	1736
Age, years			
Range	50–90	50–85	60–85
mean ± SD	71.2 (± 7.8)	70.6 (± 7.4)	73.0 (± 6.2)
Sex			
Female	52.3%	51.5%	57.3%
Male	47.7%	48.5%	42.7%
Race^b^, %			
White	76.8%	89.8%	91.5%
Asian	17.0%	8.9%	6.0%
Black	2.5%	0.8%	2.3%
Other	3.6%	0.4%	0.2%
Entry diagnosis			
Mild cognitive impairment	38.2%	81.6%	16.3%
Dementia	61.8%	18.4%	83.7%
Baseline MMSE (mean ± SD)	25.5 (± 2.2)	26.3 (± 1.7)	22.7 (± NR)
Follow‐up duration	18 months	18 months	18 months
Outcome 1^c^	CDR‐SB	CDR‐SB	CDR‐SB
Change on placebo (95% CI)	1.66 (NR)	1.74 (1.52–1.96)	2.42 (2.24–2.60)
Main effect (95% CI)	−0.45 (−0.67;−0.23)	−0.39 (−0.69;−0.09)	−0.70 (−0.95;−0.45)
Standardized effect^d^ (95% CI)	−0.34 (−0.50;−0.17)	−0.38 (−0.68;−0.09)	−0.34 (−0.47;−0.22)
Outcome 2	ADAS‐Cog14	ADAS‐Cog13	ADAS‐Cog13
Change on placebo (95% CI)	5.58 (NR)	5.16 (4.38–5.94)	7.05 (6.47–7.63)
Main effect (95% CI)	−1.44 (−2.27;−0.61)	−1.40 (−2.46;−0.34)	−1.35 (−2.14;−0.57)
Standardized effect^d^ (95% CI)	−0.19 (−0.30;−0.08)	−0.21 (−0.37;−0.05)	−0.15 (−0.24;−0.06)
Indications for unanticipated research center visit^∫^	Any ARIA‐E Symptomatic ARIA‐H	Any ARIA‐E Symptomatic ARIA‐H ≥1 superficial siderosis	Any ARIA
Indications for additional dose suspension or discontinuation^∫^	Symptomatic ARIA‐H Any macrohemorrhage >10 microhemorrhage symptomatic mild ARI‐AE Moderate ARIA‐E Severe ARIA‐E	Symptomatic ARIA‐H Any macrohemorrhage >4 microhemorrhage ≥2 superficial siderosis symptomatic mild ARIA‐E Moderate ARIA‐E Severe ARIA‐E	Symptomatic ARIA‐H Any macrohemorrhage >10 microhemorrhage >2 superficial siderosis Moderate ARIA‐E^e^ Severe ARIA‐E

Abbreviations: ADAS‐Cog, Alzheimer's Disease Assessment Scale Cognitive subscale; ARIA, amyloid‐related imaging abnormalities; ARIA‐E, amyloid‐related imaging abnormalities edema; ARIA‐H, amyloid‐related imaging abnormalities hemorrhage; CDR‐SB, Clinical Dementia Rating Sum of Boxes; CI, confidence interval; MMSE, Mini‐Mental State Examination; NR, not reported; SD, standard deviation.

^a^ Only the 1095 patients on placebo or high‐dose aducanumab are included in this table, as low‐dose treatment was not efficacious.

^b^ Among those with available data in the trial.

^c^ The CDR‐SB was the primary outcome in EMERGE and CLARITY‐AD and a secondary outcome in TRAILBLAZER‐ALZ‐2.

^d^ Standardized outcome measure (Cohen *d*).

^e^ Combined with > 4 microhemorrhages or ≥1 area of superficial siderosis; ^∫^ any single criterion by itself suffices.

Criteria for treatment suspension or dose modification were broadly similar for CLARITY‐AD and EMERGE, with additional safety MRI occurring in the case of any ARIA‐E and symptomatic ARIA‐H (Table 1). For TRAILBLAZER‐ALZ‐2, asymptomatic ARIA‐H also led to additional, unscheduled brain MRI. Based on the trial safety protocols, dose suspension and/or discontinuation were required in all trials in the case of any symptomatic ARIA, multiple microhemorrhages (>10 in CLARITY‐AD and TRAILBLAZER‐ALZ‐2, and >4 in EMERGE), or moderate to severe ARIA‐E. In TRAILBLAZER‐ALZ‐2, moderate ARIA‐E had implications only when accompanied by >4 microhemorrhages or at least one area of superficial siderosis. Similar safety measures were in place for participants in EMERGE and CLARITY‐AD who developed any macrohemorrhage (>10 mm), and for those in EMERGE and TRAILBLAZER‐ALZ‐2 who developed ≥2 areas of superficial siderosis (Table 1).

### 3.1 Incidence of adverse events

Incidence of adverse events across trials is presented in Table 2. Incidence of any type of ARIA with active treatment exceeded one in five in all trials, but with substantial heterogeneity among trials, due to variation both in occurrence of ARIA‐E and ARIA‐H. As expected, incidence of symptomatic and severe ARIA was lower than for asymptomatic and mild to moderate ARIA. Incidence of infusion‐related reaction was 26% on treatment with lecanemab, compared to 9% with donanemab.

**TABLE 2:** Incidence of adverse events in the included randomized controlled trials.

	CLARITY‐AD		EMERGE		TRAILBLAZER‐ALZ‐2	
	Lecanemab (*n* = 898)	Placebo (*n* = 897)	Aducanumab (*n* = 544)	Placebo (*n* = 541)	Donanemab (*n* = 874)	Placebo (*n* = 853)
Any ARIA	21.5%	9.5%	44%^d^	8.8%	36.8%	14.9%
ARIA‐E	12.6%	1.7%	34.8%	2.4%	24.0%	1.9%
ARIA‐H^a^	17.3%	9.0%	20.0%	6.8%	19.7%	7.4%
Superficial siderosis	5.6%	2.3%	13.5%	2.6%	6.8%	1.1%
Symptomatic ARIA^b^	3.5%	0.2%	8.8%	0.4%	6.1%	0.1%
Serious ARIA^c^	6.9%	2.9%	1.5%	0.2%	1.6%	0%
Infusion‐related reaction	26.4%	7.4%	NR	NR	8.7%	0.5%
Serious adverse event	14.0%	11.3%	13.5%	14.9%	17.4%	15.8%

Abbreviation: ARIA, amyloid‐related imaging abnormalities; ARIA‐E, amyloid‐related imaging abnormalities edema; ARIA‐H, amyloid‐related imaging abnormalities hemorrhage; NR, not reported.

^a^ For EMERGE includes microhemorrhage only, as we could not determine from the trial report the non‐overlapping numbers of patients with either microhemorrhage or superficial siderosis.

^b^ Referring only to symptomatic ARIA‐E for donanemab.

^c^ Leading to discontinuation of the trial agent (for CLARITY‐AD this is *any* adverse event, whereas for aducanumab and donanemab this refers to *serious ARIA* only).

^d^ Reflecting the percentage reported in the original trial report, even though this did not add up with the absolute numbers in that same report.

Based on trial safety protocols, ≈70% (CLARITY‐AD and EMERGE) to 100% (TRAIL‐BLAZER‐ALZ‐2) of all ARIA are estimated to have led to unblinding of trial participants. In most instances, unblinding would have been the consequence of additional safety visits including unscheduled brain MRI (Table 1). Yet, treatment suspension occurred relatively commonly too, reflected in Table 2 notably, but not exclusively, by the superficial siderosis, combined ARIA‐E/ARIA‐H, symptomatic ARIA, and severe ARIA. In the absence of severity classifications of ARIA‐E, we were unable to determine the precise share of ARIA‐E that led to treatment suspension. The severity and implications of infusion‐related reactions were not explicitly mentioned in trial reports, but based on US Food and Drug Administration documents infusion‐related reactions were moderate to severe in 31% of events, with symptoms including fever, chills, generalized aches, joint pain, nausea, vomiting, blood pressure fluctuations, and oxygen desaturation. We had no data on the frequency of notable changes to infusion rates, or initiation of adjuvant (prophylactic) therapy with, for example, antihistamines, non‐steroidal anti‐inflammatory drugs, or corticosteroids.^6^

### 3.2 Simulation results

**FIGURE 1:**
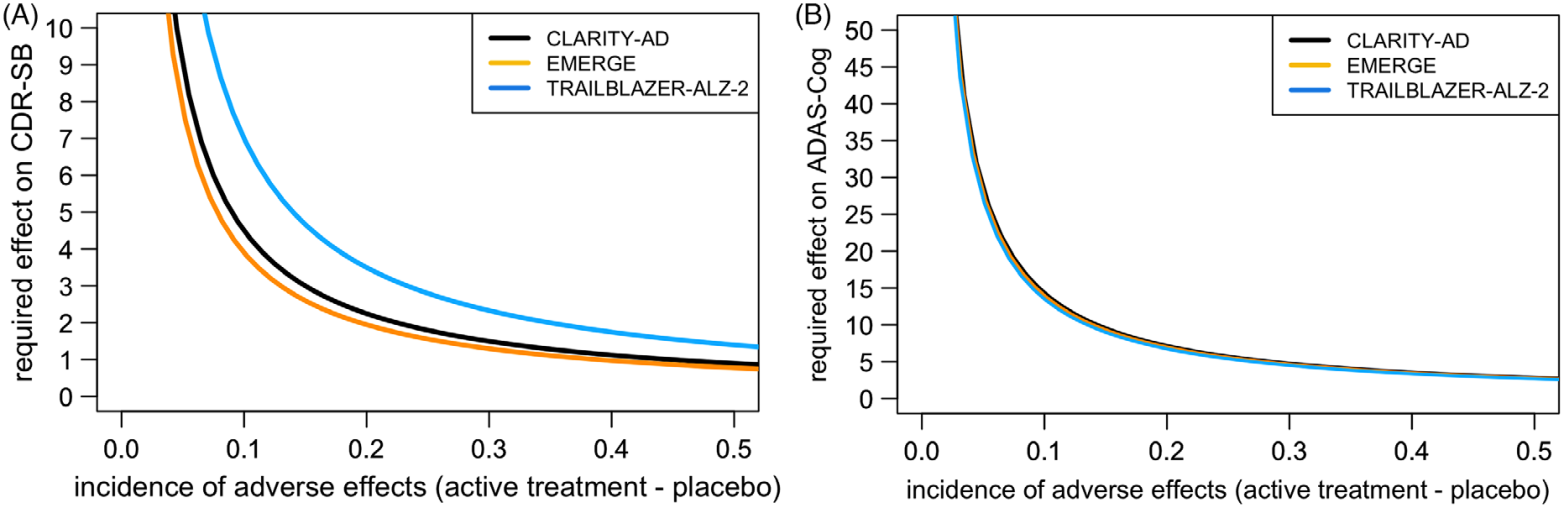
Effects of unblinding needed to fully explain observed treatment effects in different clinical trials required effects of therapeutic insight on the CDR‐SB (A), and the ADAS‐Cog (B), as a function of the incidence of adverse effects (i.e., the difference in the incidence of adverse effects between the active treatment and placebo groups) and the observed treatment effect in the trial (the colored lines). The effect estimates on the y axis reflect the magnitude of psychological treatment benefit required to explain away the observed difference in cognitive decline between active treatment and placebo groups in three different clinical trials. ADAS‐Cog, Alzheimer's Disease Assessment Scale Cognitive subscale; CDR‐SB, Clinical Dementia Rating Sum of Boxes.

The required magnitude of the psychological treatment effect required to explain away the presumed treatment effects in each of the trials is presented in Figure 1, as a function of the observed effect size per trial and the difference in incidence of ARIA between active treatment and placebo groups. With a small difference in the occurrence of adverse events between active treatment and placebo, the required magnitude quickly becomes large, due to dividing by a number closer and closer to zero. For very common adverse effects, small psychological treatment effects may suffice. The different lines for the three different trials in Figure 1 illustrate the impact of the observed treatment effect size in the trial.

**FIGURE 2:**
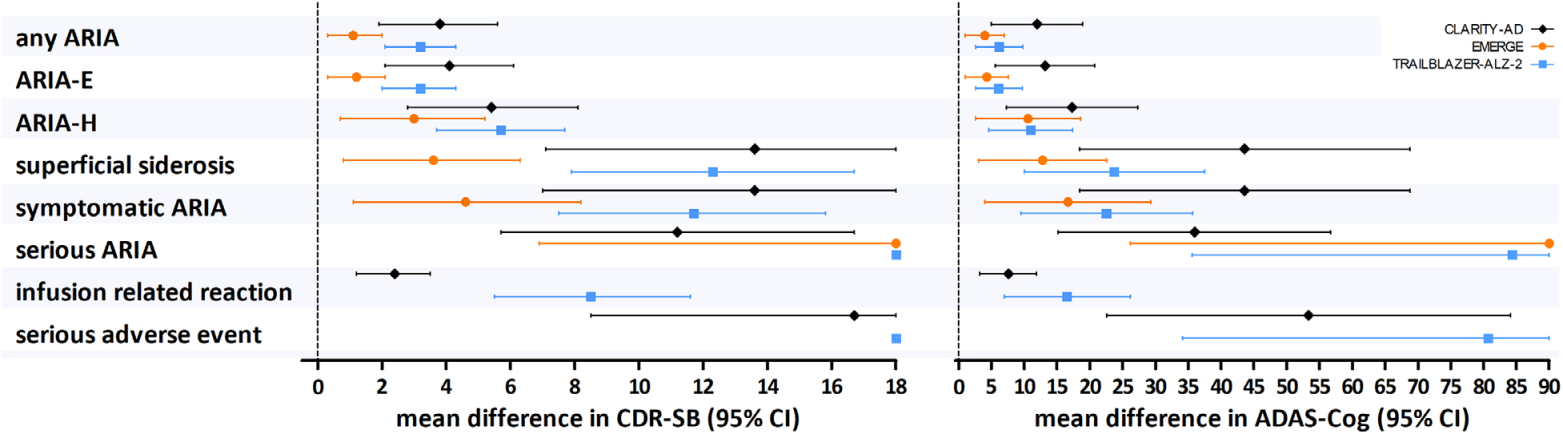
Effects of unblinding needed to explain observed treatment effects for different adverse effects. Magnitude of psychological treatment benefit required to explain away the observed difference in cognitive decline between active treatment and placebo groups in three different clinical trials, assuming the adverse effect would lead to complete unblinding. CI = confidence interval, i.e., the required magnitude of the psychological effect that corresponds to the lower and upper limit of the observed treatment effect in the RCTs. Estimates and confidence intervals were capped at the worst possible score on the cognitive outcome measures; data were not available on infusion‐related reactions for EMERGE, and for concurrent ARIA‐E and ARIA‐H for TRAILBLAZER‐ALZ‐2. Estimates for serious adverse events in EMERGE were not estimable, as they were more common with placebo than with active treatment; the ADAS‐Cog refers to the ADAS‐cog14 in EMERGE and TRAILBLAZER‐ALZ‐2 and ADAS‐cog13 in CLARITY‐AD. ADAS‐Cog, Alzheimer's Disease Assessment Scale Cognitive subscale; ARIA, amyloid‐related imaging abnormalities; ARIA‐E, amyloid‐related imaging abnormalities edema; ARIA‐H, amyloid‐related imaging abnormalities hemorrhage; CDR‐SB, Clinical Dementia Rating Sum of Boxes; RCT, randomized controlled trial.

Presuming all ARIA led to unblinding due to unplanned MRI, the magnitude of the psychological treatment effect that is required to fully explain the observed treatment effect on the CRD‐SB in the RCTs ranged from 1.1 (95% confidence interval [CI]: 0.2–2.0) in EMERGE, to 3.2 (2.1–4.3) in TRAILBLAZER‐ALZ‐2 and 3.8 (1.9–5.6) in CLARITY‐AD (Figure 2, Table S1 in supporting information). Estimates were generally smallest in EMERGE, followed by CLARITY‐AD and TRAILBLAZER‐ALZ‐2. For adverse effects that prompted treatment suspension, most effect estimates were large. For example, CDR‐SB estimates for superficial siderosis ranged from 3.6 in EMERGE to 13.9 in CLARITY‐AD (Figure 2). Estimates for infusion‐related reactions were substantially higher in CLARITY‐AD than in TRAILBLAZER‐ALZ‐2 (for CDR‐SB: 2.4 [1.2–3.5] vs. 8.5 [5.5–11.6]), due to their higher reported incidence in CLARITY‐AD (Figure 2). Standardized effect estimates (Cohen *d*) are presented in Table S2 in supporting information.

Results for the ADAS‐Cog were consistent with those for the CDR‐SB (Figure 2, Tables S1 and S2). For example, the magnitude of the psychological treatment effect required to fully explain the observed treatment effect by ARIA incidence, ranged from 4.0 (95% CI: 1.0–7.0) in EMERGE, to 6.2 (2.6–9.8) in TRAILBLAZER‐ALZ‐2 and 12.0 (5.1–18.9) in CLARITY‐AD. Here, the difference between RCTs was driven chiefly by the incidence of adverse events, as observed treatment effects on the ADAS‐Cog were more similar across trials than for the CDR‐SB.

## 4 DISCUSSION

Based on published results from RCTs, we provide estimates for the magnitude of unblinding effects needed to explain the observed treatment effects of monoclonal antibodies against Aβ. In the following paragraphs, we will discuss the implications of these estimates, including the degree of unblinding across trials, the share of observed treatment effects that can reasonably be explained by unblinding, and the implications for trial design and estimated drug efficacy.

The precise impact of adverse effects on blinding to treatment allocation is unknown, but on the basis of the trial protocols,^1^, ^2^, ^3^ we determined that 70% to 100% of ARIA lead to some degree of therapeutic insight. Until now, investigators of the RCTs of monoclonal antibodies against Aβ have attempted to address unblinding in the included trials through sensitivity analyses that censor study participants at the time of the unblinding event, which showed minimal change—if any—in the observed treatment effect. This censoring method has limitations, as it excludes trial participants with the most side effects and causes imbalance between the active treatment and placebo groups, which biases results in favor of active treatment (unless the incidence of adverse events is not related to any other cause of the outcome). Future RCTs could specify strategies to prevent unblinding due to adverse events, for example by inviting participants on placebo for additional imaging whenever a participant on active treatment develops side effects that warrant deviation from the standard treatment.^5^ Perhaps the most straightforward way of discerning the effects of unblinding is to ask participants about their perceived treatment allocation at the end of the trial.^7^ Although methods have been developed for doing so,^8^ the caregiver input on the CDR‐SB may warrant methods tailored to dementia research. Moreover, it should be noted that end‐of‐trial tests for unblinding cannot distinguish unblinding from hunches about treatment efficacy (caused by the clinical progression along the course of the trial).^9^, ^10^ For this reason, the Consolidated Standards of Reporting Trials statement does not provide investigators with recommendations on the assessment of blinding.^10^

No published studies have specifically assessed the placebo effect on any of the cognitive outcome measures used in the trials of monoclonal antibodies against AD. Borrowing from other fields of medicine, treatment response to placebo has been reported in 16% of patients in Parkinson's disease trials,^11^ and in up to 20% of patients in trials against epilepsy.^12^ In a systematic review of clinical depression treatment trials, the pooled average placebo effect had a magnitude *g* of 1.05, with largest effects in more recent studies and industry‐sponsored trials.^13^ This standardized effect *g* is equivalent to approximately 1 to 2 points on the CDR‐SB, and ≈ 6 to 9 points on the ADAS‐Cog. Although caution is warranted in extrapolating findings from placebo effects on other outcomes to unblinding effects on cognitive outcome measures, these reports suggest that the effect estimates in our analyses could explain a substantial share of the observed treatment effects. Extrapolating these standardized effects, for example, to incidence of ARIA‐E with lecanemab (Table S1), this could imply 25% to 50% of observed treatment effects may be explained by unblinding. The magnitude of other partial effects can be easily derived from our tables and figures; for example, to determine the effect needed to explain half of the observed treatment effect, simply divide the reported estimates by two. Future research could serve to quantify the psychological treatment effect on cognitive outcome measures, and determine whether effects of unblinding due to adverse effects are in fact comparable to placebo effects.

The results of this simulation rely on several assumptions and considerations. First, there was substantial heterogeneity between trials in the incidence of adverse effects, which may be attributable to eligibility criteria, pharmacokinetics, and pharmacodynamics, but could also reflect variation in the definition and adjudication of, for example, infusion‐related reactions. Associated symptoms can be underreported by patients too, as those who suspect they are on active treatment may downplay or deny symptoms. Systematic inquiry of ARIA‐related symptoms may in part alleviate this concern. Second, the share of adverse events that led to unblinding was not always clear from trial reports. In particular, unscheduled MRI could have been performed at the investigators’ discretion. We also could not fully determine the share of moderate ARIA‐E that led to treatment suspension, in addition to the additional MRI visits. Prior reports suggest that ≈ 50% of all ARIA‐E on treatment with aducanumab and lecanemab are of moderate severity.^14^, ^15^ Third, we assumed that any modification to the standard trial protocol due to adverse events would have similar effects on therapeutic insight, in the active treatment as well as placebo groups, while additional research visits and treatment suspension may differentially affect patient perception. Fourth, trial reports did not allow estimation of the joint, cumulative burden of any potentially unblinding event, rather than separate events in isolation. Fifth, our estimates do not account for effects of unblinding on differential attrition. If patients and caregivers aware of allocation to the experimental drug are less inclined to withdraw from the study in the case of a relatively favorable disease course, as opposed to a period with rapid decline, this too may hamper internal validity of the trial and amplify the effects on unblinding above and beyond the estimates described in this report. Finally, the part of the observed treatment effect that is *not* due to unblinding also includes potential detrimental effects of ARIA on cognition (e.g., due to macrohemorrhage). If such effects are larger than the positive effect of therapeutic insight, the impact of unblinding is negligible.

In conclusion, unblinding to treatment allocation due to adverse effects in RCTs against AD is unlikely to fully account for observed treatment effects in CLARITY‐AD and TRAILBLAZER‐ALZ‐2, but may explain a substantial share of the observed treatment effects in all published trials that reported efficacy of Aβ‐removing therapy. Future RCTs could take precautionary measures to prevent unblinding or mitigate its effects at the trial's end. Meanwhile, these results warrant consideration when evaluating trial efficacy and clinical relevance of the observed treatment effects.

## CONFLICT OF INTEREST STATEMENT

The authors declare no conflicts of interest. Author disclosures are available in the supporting information.

## CONSENT STATEMENT

No informed consent was required as no individual participant data were used for this report.

## Supporting information

Supplementary Tables

Supporting Information
