# Supplementary material for: Potential impact of unblinding on observed treatment effects in Alzheimer's disease trials
Source: Alzheimers Dement. 2024 Feb 21;20(4):3119–25. doi: 10.1002/alz.13690 (PMC11032516; doi:10.1002/alz.13690)
Supplement: Supplementary file 1 — Supplementary Tables [file ALZ-20-3119-s001.docx]

Online supplement with

**Potential impact of unblinding on observed treatment effects in Alzheimer’s disease trials**

Frank J. Wolters, MD PhD^1,2^

Jeremy A. Labrecque, PhD^1^

**Supplementary Table S1. Required effect of therapeutic insight on cognitive outcome**

Legend: Magnitude of psychological treatment benefit required to explain away the observed difference in cognitive decline between active treatment and placebo groups in clinical trials. Data are presented graphically in Figure 2.

|  | **CLARITY-AD** | **EMERGE** | **TRAILBLAZER-ALZ-2** |
| --- | --- | --- | --- |
|  | mean difference (95% CI) | mean difference (95% CI) | mean difference (95% CI) |
|  |  |  |  |
| **CDR-SB** |  |  |  |
| Any ARIA | -3.8 (-5.6;-1.9) | -1.1 (-2.0;-0.3) | -3.2 (-4.3;-2.1) |
| ARIA-E | -4.1 (-6.1;-2.1) | -1.2 (-2.1;-0.3) | -3.2 (-4.3;-2.0) |
| ARIA-H | -5.4 (-8.1;-2.8) | -3.0 (-5.2;-0.7) | -5.7 (-7.7;-3.7) |
| Superficial siderosis | -13.6 (-18.0;-7.0) | -3.6 (-6.3;-0.8) | -12.3 (-16.7;-7.9) |
| Symptomatic ARIA | -13.6 (-18.0;-7.0) | -4.6 (-8.2;-1.1) | -11.7 (-15.8;-7.5) |
| Serious ARIA | -11.2 (-16.7;-5.7) | -18.0 (-18.0;-6.9) | -18.0 (-18.0;-18.0) |
| Infusion related reaction | -2.4 (-3.5;-1.2) | NA | -8.5 (-11.6;-5.5) |
| Serious adverse event | -16.7 (-18.0;-8.5) | NE | -18.0 (-18.0;-18.0) |
|  |  |  |  |
| **ADAS-Cog^*^** |  |  |  |
| Any ARIA | -12.0 (-18.9;-5.1) | -4.0 (-7.0;-1.0) | -6.2 (-9.8;-2.6) |
| ARIA-E | -13.2 (-20.8;-5.6) | -4.3 (-7.6;-1.0) | -6.1 (-9.7;-2.6) |
| ARIA-H | -17.3 (-27.3;-7.3) | -10.6 (-18.6;-2.6) | -11.0 (-17.4;-4.6) |
| Superficial siderosis | -43.6 (-68.8;-18.5) | -12.8 (-22.6;-3.1) | -23.7 (-37.5;-10.0) |
| Symptomatic ARIA | -43.6 (-68.8;-18.5) | -16.7 (-29.3;-4.0) | -22.5 (-35.7;-9.5) |
| Serious ARIA | -36.0 (-56.7;-15.2) | -90.0 (-90.0;-26.2) | -84.4 (-90.0;-35.6) |
| Infusion related reaction | -7.6 (-11.9;-3.2) | NA | -16.5 (-26.2;-7.0) |
| Serious adverse event | -53.3 (-84.1;-22.6) | NE | -80.7 (-90.0;-34.1) |
|  |  |  |  |

CI=confidence interval, i.e., the required magnitude of the psychological effect that corresponds to the lower and upper limit of the observed treatment effect in the RCTs. Estimates and confidence intervals were capped at the worst possible score on the cognitive outcome measures; NA=information not available; NE=not estimable, as serious adverse events were more common with placebo than with active treatment; ^*^ ADAS-cog14 in EMERGE and TRAILBLAZER-ALZ-2 and ADAS-cog13 in CLARITY-AD

**Supplementary Table S2. Required effect of therapeutic insight on cognitive outcome, in standard deviations**

Legend: Analyses similar to supplementary table S1, but expressed as standard devisations of the particular outcome measure (Cohen’s d). Estimates thus reflect the magnitude of psychological treatment benefit in standard deviations required to explain away the observed difference in cognitive decline between active treatment and placebo groups in clinical trials.

|  | **CLARITY-AD** | **EMERGE** | **TRAILBLAZER-ALZ-2** |
| --- | --- | --- | --- |
|  | mean difference (95% CI) | mean difference (95% CI) | mean difference (95% CI) |
|  |  |  |  |
| **CDR-SB** |  |  |  |
| Any ARIA | -2.8 (-4.2;-1.4) | -1.1 (-1.9;-0.3) | -1.5 (-2.1;-1.0) |
| ARIA-E | -3.1 (-4.6;-1.6) | -1.2 (-2.1;-0.3) | -1.5 (-2.0;-1.0) |
| ARIA-H | -4.0 (-6.0;-2.1) | -2.9 (-5.1;-0.7) | -2.7 (-3.7;-1.7) |
| Superficial siderosis | -10.2 (-15.2;-5.2) | -3.5 (-6.2;-0.8) | -5.8 (-7.9;-3.8) |
| Symptomatic ARIA | -10.2 (-15.2;-5.2) | -4.6 (-8.1;-1.1) | -5.6 (-7.5;-3.6) |
| Serious ARIA | -8.4 (-12.5;-4.3) | -17.6 (-17.6;-6.8) | -8.6 (-8.6;-8.6) |
| Infusion related reaction | -1.8 (-2.6;-0.9) | NA | -4.1 (-5.5;-2.6) |
| Serious adverse event | -12.4 (-13.4;-6.4) | NE | -8.6 (-8.6;-8.6) |
|  |  |  |  |
| **ADAS-Cog^*^** |  |  |  |
| Any ARIA | -1.6 (-2.6;-0.7) | -0.6 (-1.0;-0.1) | -0.7 (-1.1;-0.3) |
| ARIA-E | -1.8 (-2.8;-0.8) | -0.6 (-1.1;-0.2) | -0.7 (-1.1;-0.3) |
| ARIA-H | -2.4 (-3.7;-1.0) | -1.5 (-2.7;-0.4) | -1.2 (-2.0;-0.5) |
| Superficial siderosis | -6.0 (-9.4;-2.5) | -1.9 (-3.3;-0.5) | -2.7 (-4.2;-1.1) |
| Symptomatic ARIA | -6.0 (-9.4;-2.5) | -2.4 (-4.3;-0.6) | -2.5 (-4.0;-1.1) |
| Serious ARIA | -4.9 (-7.7;-2.1) | -13.1 (-13.1;-3.8) | -9.5 (-10.2;-4.0) |
| Infusion related reaction | -1.0 (-1.6;-0.4) | NA | -1.9 (-2.9;-0.8) |
| Serious adverse event | -7.3 (-11.5;-3.1) | NE | -9.5 (-10.2;-4.0) |
|  |  |  |  |

CI=confidence interval, i.e., the required magnitude of the psychological effect that corresponds to the lower and upper limit of the observed treatment effect in the RCTs. Estimates and confidence intervals were capped at the worst possible score on the cognitive outcome measures; NA=information not available; NE=not estimable, as serious adverse events were more common with placebo than with active treatment; ^*^ ADAS-cog14 in EMERGE and TRAILBLAZER-ALZ-2 and ADAS-cog13 in CLARITY-AD
